# Supplementary material for: Death toll among the Bangladeshi refugees of the 1971 war
Source: PLoS One. 2025 Apr 4;20(4):e0320760. doi: 10.1371/journal.pone.0320760 (PMC11970699; doi:10.1371/journal.pone.0320760)
Supplement: S1 Text — (DOCX) [file pone.0320760.s001.docx]

**S1 Text. Refugee influx over time**

Refugee numbers were aggregated by the Govt. of India, and published in (Ministry of External Affairs, India, 1971) (Volume 1, page 446,461-462, and Volume 2, page 81-82).

Total monthly refugee numbers can be calculated from the monthly influx numbers provided by the Govt. of India (Ministry of External Affairs, India, 1971)(Volume 2, page 81-82):

| **Date** | **Total Influx** |
| --- | --- |
| 10-Apr | 0 |
| 30-Apr | 1,221,000 |
| 31-May | 4,379,000 |
| 30-Jun | 6,435,000 |
| 31-Jul | 7,232,000 |
| 31-Aug | 8,287,000 |
| 30-Sep | 9,091,000 |
| 31-Oct | 9,516,000 |
| 30-Nov | 9,733,000 |
| 15-Dec | 9,899,305 |

Total weekly refugee numbers can be calculated from the weekly influx numbers provided by the Govt. of India (Ministry of External Affairs, India, 1971)(Volume 1, page 446):

| **Date** | **Total Influx** |
| --- | --- |
| 10-Apr | 0 |
| 17-Apr | 119,566 |
| 24-Apr | 655,874 |
| 1-May | 867,428 |
| 8-May | 1,572,220 |
| 15-May | 2,399,667 |
| 22-May | 3,371,931 |
| 29-May | 3,688,350 |
| 5-Jun | 4,982,792 |
| 12-Jun | 5,767,172 |
| 19-Jun | 5,923,439 |
| 26-Jun | 6,325,998 |
| 3-Jul | 6,541,446 |
| 10-Jul | 6,733,076 |
| 17-Jul | 7,021,490 |
| 24-Jul | 7,058,826 |
| 31-Jul | 7,133,004 |
| 7-Aug | 7,364,979 |
| 14-Aug | 7,567,257 |
| 21-Aug | 8,018,743 |
| 28-Aug | 8,256,804 |
| 31-Aug | 8,281,220 |

Also, during this period, refugee numbers were reported occasionally in newspapers quoting national or state govt. officials (Mamoon & Haq, 2007). Refugee numbers for various dates collected from these sources is presented in the table below.

| **Date** | **Total Influx** | **Reference** |
| --- | --- | --- |
| 21-Apr | 318,000 | Volume 11, page 112, (Mamoon & Haq, 2007) |
| 24-Apr | 495,000 | Volume 18, page 15, (Mamoon & Haq, 2007) |
| 23-May | 3,200,000 | Volume 6, page 96, (Mamoon & Haq, 2007) |
| 25-May | 3,500,000 | Volume 11, page 114-15, (Mamoon & Haq, 2007) |
| 29-May | 3,780,134 | Volume 18, page 22, (Mamoon & Haq, 2007) |
| 3-Jun | 4,730,517 | Volume 1, page 461, (Ministry of External Affairs, India, 1971) |
| 8-Jun | 5,441,683 | Volume 18, page 56, (Mamoon & Haq, 2007) |
| 10-Jun | 5,765,000 | Volume 11, page 128-29, (Mamoon & Haq, 2007) |
| 15-Jun | 5,800,000 | Volume 1, page 462, (Ministry of External Affairs, India, 1971) |
| 16-Jun | 5,767,000 | Volume 18, page 62-63, (Mamoon & Haq, 2007) |
| 22-Jun | 6,002,400 | Volume 18, page 79, (Mamoon & Haq, 2007) |
| 14-Jul | 6,833,000 | Volume 18, page 87-88, (Mamoon & Haq, 2007) |
| 17-Jul | 7,021,524 | Volume 18, page 92, (Mamoon & Haq, 2007) |
| 27-Jul | 6,964,000 | Volume 6, page 105, (Mamoon & Haq, 2007) |
| 1-Sep | 8,281,220 | Volume 18, page 98-99, (Mamoon & Haq, 2007) |
| 24-Sep | 8,962,000 | Volume 18, page 109-110, (Mamoon & Haq, 2007) |
| 20-Oct | 9,300,000 | Volume 18, page 119, (Mamoon & Haq, 2007) |
| 4-Nov | 9,576,000 | Volume 18, page 126, (Mamoon & Haq, 2007) |
| 28-Nov | 9,733,000 | Volume 18, page 137, (Mamoon & Haq, 2007) |
| 7-Dec | 9,771,000 | Volume 18, page 138-9, (Mamoon & Haq, 2007) |
| 9-Dec | 9,800,000 | Volume 18, page 139, (Mamoon & Haq, 2007) |

These 3 sets of numbers are plotted together in Figure 1.

However, the official influx data is not perfect and contains some oddities. For example, the total count on 31^st^ August 1971 differs between the two volumes of (Ministry of External Affairs, India, 1971), one being 8,281,220 and another 8,287,000.

The start date of influx records also varies among sources. The records presented in (Ministry of External Affairs, India, 1971) seem to begin on 10^th^ April 1971. However, refugee influx began right after the Operation Searchlight on 25^th^ March, and by 5^th^ April a newspaper report already mentions 5,927 refugees in the state of Tripura (Volume 22, Page 75, (Mamoon & Haq, 2007)). We take the later date of 10^th^ April, as officially reported by the govt. publication. It also means the total contribution to the estimated death count will be slightly lower, making it a slight underestimate in this regard. We removed any unusual numbers, such as duplicates, or entries that give a far lower total population than an earlier date.

Sometimes there were unofficial estimates of refugee numbers, which were higher than the official counts. For example, on 29^th^ April the official count for West Bengal was 673,000, but an unofficial figure was 2 million (Volume 6, Page 90, (Mamoon & Haq, 2007)). In some cases, the unofficial figures were impossibly high, such as an estimate of 10 million for entire India on 27^th^ April (Volume 6, Page 89, (Mamoon & Haq, 2007). We do not use the unofficial figures here. Even though Pakistan officially disputed the Indian refugee count and suggested a smaller figure, independent observers concluded the Indian figure to be mostly reliable (S4 Text).

The aforementioned three sets of influx numbers are organized into the table below.

| **Row** | **Date** | **Date (numerical)** | **Date difference** | **Population** | **Area (person-time)  (million people × day)** |
| --- | --- | --- | --- | --- | --- |
| 1 | 10-Apr | 0 | — | 0 | — |
| 2 | 17-Apr | 7 | 7 | 0.119566 | 0.418481 |
| 3 | 24-Apr | 14 | 7 | 0.655874 | 2.714040 |
| 4 | 30-Apr | 20 | 6 | 1.221000 | 5.630622 |
| 5 | 8-May | 28 | 8 | 1.572220 | 11.172880 |
| 6 | 15-May | 35 | 7 | 2.399667 | 13.901605 |
| 7 | 22-May | 42 | 7 | 3.371931 | 20.200593 |
| 8 | 29-May | 49 | 7 | 3.688350 | 24.710984 |
| 9 | 31-May | 51 | 2 | 4.379000 | 8.067350 |
| 10* | 5-Jun | 56 | 5 | 4.982792 | 23.404480 |
| 11* | 12-Jun | 63 | 7 | 5.767172 | 37.624874 |
| 12* | 19-Jun | 70 | 7 | 5.923439 | 40.917139 |
| 13* | 26-Jun | 77 | 7 | 6.325998 | 42.873030 |
| 14* | 30-Jun | 81 | 4 | 6.435000 | 25.521996 |
| 15* | 3-Jul | 84 | 3 | 6.541446 | 19.464669 |
| 16* | 10-Jul | 91 | 7 | 6.733076 | 46.460827 |
| 17* | 17-Jul | 98 | 7 | 7.021490 | 48.140981 |
| 18* | 24-Jul | 105 | 7 | 7.058826 | 49.281106 |
| 19* | 31-Jul | 112 | 7 | 7.232000 | 50.017891 |
| 20* | 7-Aug | 119 | 7 | 7.364979 | 51.089427 |
| 21* | 14-Aug | 126 | 7 | 7.567257 | 52.262826 |
| 22* | 21-Aug | 133 | 7 | 8.018743 | 54.551000 |
| 23* | 28-Aug | 140 | 7 | 8.256804 | 56.964415 |
| 24* | 31-Aug | 143 | 3 | 8.287000 | 24.815706 |
| 25* | 30-Sep | 173 | 30 | 9.091000 | 260.670000 |
| 26 | 31-Oct | 204 | 31 | 9.516000 | 288.408500 |
| 27 | 30-Nov | 234 | 30 | 9.733000 | 288.735000 |
| 28 | 15-Dec | 249 | 15 | 9.899305 | 147.242288 |
| 29 | 31-Dec | 265 | 16 | 9.899305 | 158.388880 |

To calculate the total person-time, we follow standard demographic convention, in which the value for any particular interval of time is taken to correspond to the middle of the period (Ramakumar & Gopal, 1986). Numerically, this is equivalent to calculating the area under the curve by the trapezoid rule. For example, the total population was 0.119566 million on 17 April and 0.655874 million on 24 April. Therefore, the duration of this period is 7 days, and the average population is (0.119566 + 0.655874)/2. Therefore, the total area of this trapezoid, i.e. the total person-time in this period, is 7 × (0.119566 + 0.655874)/2 = 2.714040, which is given in the last column.

The total of all these person-time values is 1853.65 million people × day, which is the total person-time spent by all refugees in India.

As the monsoon season was taken to be from June to September (S2 Text), rows 10-25 (marked by *) indicate those periods that fall within the monsoon. Therefore, the areas corresponding to these rows can be aggregated to 884.06 million people × day, which is the total person-time spent by all refugees in India during the monsoon season of June-September.

# References

Mamoon, M., & Haq, A. M. (2007). *Media and the Liberation War of Bangladesh.* Dhaka: Ananya.

Ministry of External Affairs, India. (1971). *Bangla Desh Documents.* New Delhi: Ministry of External Affairs.

Ramakumar, R., & Gopal, Y. S. (1986). *Technical Demography.* New Delhi: Wiley Eastern Limited.
