# Supplementary material for: Death toll among the Bangladeshi refugees of the 1971 war
Source: PLoS One. 2025 Apr 4;20(4):e0320760. doi: 10.1371/journal.pone.0320760 (PMC11970699; doi:10.1371/journal.pone.0320760)
Supplement: S2 Text — (DOCX) [file pone.0320760.s002.docx]

**S2 Text: The monsoon season in India during 1971**


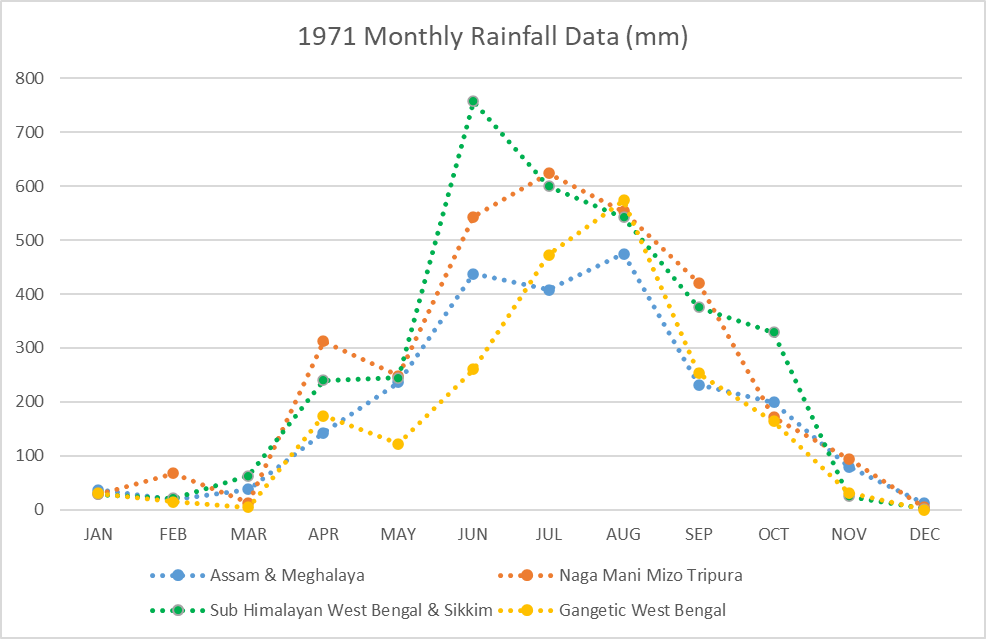


We tracked the monsoon period in 1971 using historical data from the India Meteorological Department (India Meteorological Department (IMD), 2019), a branch of the Government of India. Aggregate monthly rainfall data (in mm) was available for the 36 meteorological subdivisions of India (India Meteorological Department (IMD), 2019), of which the four relevant areas covering Eastern India were used. The rainfall data, as shown in the figure above, show a prominent increase during the period of June to September, which is denoted as the monsoon period by IMD. It is important to note that monsoon arrived earlier in the hill areas of eastern India (such as the states of Meghalaya), in May, whereas it arrived at the Gangetic plains of West Bengal in June. This is also supported by reports from refugee camps (Pages 72-73, (Chaudhuri, 1972)), which mentions that floods and cholera started in May among the refugee camps in hill areas, whereas the full extent of cholera in Gangetic plains of West Bengal started in early June. The monsoon floods continued with full severity till the end of September, including cyclones sweeping through Eastern India (Associated Press, 1971).

It is important for this study to consider a specific period for monsoon since in our estimation procedure we are considering a monsoon-specific increase in mortality rates. Also, in the data of several camps, an explicit date of the start or end of the reporting period is not provided. In such cases, if the deaths are from monsoon-specific causes, then we consider the date to be the start or end of the monsoon period. For example, the data for the Karimpur camp, or the second data point for the Salt Lake camp, correspond to the monsoon-specific death factor, cholera. On the other hand, the deaths for the Itkhola camp were from other monsoon-specific causes such as diarrhea. So, in all these cases the starting period of the reporting date was taken to be 1^st^ June.

# References

Associated Press. (1971, November 2). Cyclone, Wave Kill Thousands in India. *Los Angeles Times*, p. A1.

Chaudhuri, K. (1972). *Genocide in Bangladesh.* Bombay: Orient Longman.

India Meteorological Department (IMD). (2019). *Area weighted monthly, seasonal and annual rainfall ( in mm) for 36 meteorological subdivisions.* Retrieved from Open Government Data (OGD) Platform India: https://data.gov.in/resources/area-weighted-monthly-seasonal-and-annual-rainfall-mm-36-meteorological-subdivisions-1901

India Meteorological Department (IMD). (2019). *Rainfall in India.* Retrieved from Open Government Data (OGD) Platform India: https://data.gov.in/catalog/rainfall-india
