# Supplementary material for: Death toll among the Bangladeshi refugees of the 1971 war
Source: PLoS One. 2025 Apr 4;20(4):e0320760. doi: 10.1371/journal.pone.0320760 (PMC11970699; doi:10.1371/journal.pone.0320760)
Supplement: S3 Text — (DOCX) [file pone.0320760.s003.docx]

**S3 Text: Reasoning why our estimate of death toll is an undercount**

**3.1. Death rates in the Northeastern States’ refugee camps were probably higher than that in West Bengal’s refugee camps**

Conditions of refugee camps of north-eastern districts such as Assam and Meghalaya were reportedly worse than those of West Bengal, and the mortality rates from various causes were higher (Page 41, (United Nations High Commissioner for Refugees, 1972)) (Schanberg, 1971). These regions had smaller populations and smaller infrastructure to begin with, and had difficulty coping up with the huge influx which was sometimes even larger than the local population. The supply of essential items was also affected due to their relative isolation from the rest of the country. As M.P. Bezbaruah, Deputy Commissioner of Goalpara district of Assam noted, the town of Dhubri had a population of 30,000 while the new refugee camp at nearby Chapor contained 50,000 refuges. It “was almost like setting up a new township.”(Page 221, (Saha, 2003)). In Tripura too, the net refugee influx was 1.35 million compared to its local population of 1.56 million (Page 218-219, (Saha, 2003)).

Antara Dutta comments (Datta, 2013)(Page 87), “Assam and West Bengal had, in the post-Partition period, very different experiences with refugees. … Assam was resistant to accepting refugees”. Datta postulates that this difference could be due to the ethnicity of the Bengali refugees being different to that of the residents of the north-eastern states.

Table 1 shows that 73% of all refugees stayed in West Bengal, while the remaining 27% stayed in the Northeastern states such as Meghalaya, Tripura, Assam, etc. Comparatively fewer data points were available on refugee mortality from these states, as can be seen in Table 2. For example, there is no information available from Tripura. Due to this scarcity of data, we could not use more complex mathematical models which could’ve allowed for separate estimation of death rates in the Northeastern states.

**3.2. Salt Lake was one of the ‘better conditioned’ refugee camps**

Our current estimate is heavily influenced by Salt Lake camp’s death toll due to its large camp population size. However, Salt Lake camp’s conditions were better than the conditions of other refugee camps of India. J. A. Seaman commented (Seaman, 1972), “Salt Lake, …, was the closest of the camps to Calcutta, the least bound by restrictions, and the most visited by the Press. It thus received a disproportionate amount of assistance. It is my assumption that conditions for refugees in other camps, especially for children, were worse”. S. M. Schanberg reported that the Salt Lake camp was a “demonstration” camp which foreign relief workers were allowed to visit, but other camps were barred to them (Schanberg, 1971). We observed in our calculations that the baseline death rate in Salt Lake was lower than the overall estimated death rate, as expected.

**3.3. Higher mortality among refugees living outside camps**

Of the 10 million refugees in India, nearly 1/3^rd^ were living outside of the camps (Table 1) (Ministry of External Affairs, India, 1971) (Volume 2, page 81). Though the official term for these people was that they were living with “relatives” or “host families” (Page 66, (United Nations High Commissioner for Refugees, 2000)), many of them were living on the streets.

On occasions, the refugees registered at a camp, but left the camp in search of better prospects. A news report in June mentions a group of 20,000 refugees who registered at the Salt Lake camp, but nearly half of them went to the nearby city of Calcutta, living on the streets as homeless (Volume 11, Page 135, (Mamoon & Haq, 2007)).

Many refugees were displaced out of the camps due to monsoon. A report from the Reuters in mid-September mentions the devastation brought about by the monsoon floods, saying that 350,000 refugees were ‘marooned’ (Reuters, 1971). A PTI report in late September mentioned 322,000 refugees from 21 camps in Krishnanagar Sadar division were displaced out of camps due to flood, and were sitting by the roads and highways with nowhere to go (PTI, 1971). It mentioned many people dying from starvation, cold, cholera, diarrhea, and blood dysentery, with the dead being buried there by their relatives without being reported to the officials.

Those living outside refugee camps were not eligible for any assistance as per Central Government rules (Page 212, (Saha, 2003)). Therefore, without access to aid and vaccinations, and living without shelters, it is likely that mortality rate among these people were higher. But there are no proper records of mortality among these people, possibly because collecting data was much more difficult.

**3.4. Undercount of deaths from malnutrition**

An aggregated estimate of death count among refugees, from a news report on 22^nd^ June 1971, surmised that approximately 300,000 people have died from various diseases, while 300,000 more have died from malnutrition (Foster, 1971). However, many of the reported death counts included in this study are primarily due to a single cause, such as cholera, or cyclone (Table 2). Other causes such as malnutrition are relatively rarely reported.

The aforementioned report put malnutrition to be a killer comparable to cholera; the high prevalence of malnutrition and it being a major factor of death were widely reported (Schanberg, 1971) (Associated Press, 1971). A refugee camp worker from a voluntary organization reported that malnutrition was so severe and widespread that “100,000 might die during the following ten days” (Page 57, (United Nations High Commissioner for Refugees, 1972)). Even after the floods receded in October and the camp conditions started to improve, Mr. Labouisse, Executive Director of UNICEF, commented after his visit to the camps: “Malnutrition among refugee children may get worse before it gets better and a few thousands deaths in the coming weeks could not be ruled out.” (Page 58, (United Nations High Commissioner for Refugees, 1972)).

Two surveys estimated that 50% (Page 27, (The Comptroller General of The United States, 1972)) and 82% (Swaminathan, Vijayaraghavan, & Rao, 1983) of the children in camps suffer from malnutrition. Bridget Battey, a nurse working for Save the Children in the ‘better conditioned’ Salt Lake camp, reported towards the end of 1971 that malnutrition was the biggest killer among children in her experience, and their overall mortality rate was as high as 15% (Page 62, (Malik, The Year of the Vulture, 1972)).

**3.4. Lack of records on births after arrival**

While the very high mortality of newborn children were often reported (Schanberg, 1971), no estimate of the number of births among the refugees are available. A news report on 5^th^ October (Brogan, 1971) suggested that the number of pregnant or breastfeeding mothers in various camps could be 500,000. Another report mentioned “two babies born recently in Gopanpal Nagar camp both died within hours” (Foster, 1971).

The refugee count by the Indian government was done at the point of entry in India, or while registering at a refugee camp, so it does not include these subsequent births. Not only these should’ve contributed to the total refugee population, these unrecorded children probably had the highest death rate (Toole & Waldman, 1988) (Mercer, 1992), thereby leading to a double underestimation of the total mortality.

**3.5. Non-recording of wayside deaths**

“Wayside side” refers to the death of refugees who could not reach a refugee camp and died on the way, which are usually not counted. Refugees making long journeys through Bangladesh and India faced the most challenging circumstances, including difficult roads and terrains (Page 72, (Chaudhuri, 1972)), monsoon rains and floods, epidemics, and firing by the Pakistan army, while suffering from exhaustion and malnutrition. Those who eventually managed to reach the camps were in a very weakened condition. In particular, children suffered the most in these arduous journeys (Page 62, (Malik, The Year of the Vulture, 1972)). A camp doctor attending the arriving children observed: “A lot of them arrive in an irreversible state – a condition of complete collapse. There's nothing we can do for them.” (Schanberg, 1971). In many cases, families were unable to carry the young children anymore, and left them on the roadside to die (Page 139, (Gerlach, 2012)) (Page 63, (Malik, The Year of the Vulture, 1972)).

As W.B. secretary to health ministry Mr. K. K. Dass admitted in June, wayside death numbers are at least as large as the counted deaths due to cholera in camps (United News of India, 1971) (Saar, 1971). Nadia district officer Mr. D. K. Ghosh mentioned in June that while official estimates of cholera deaths in his district was 4000, more than 2000 refugees died on the wayside during an influx of 400,000 from Bangladesh just in one week at the end of May. He said that Govt. officials had to collect their bodies and bury them in mass graves (Coughlin, 1971).In an interview by K. C. Saha, the additional district magistrate of Nadia mentioned the deaths of hundreds of refugees on the wayside due to exhaustion. He also mentioned about 800 refugees who died soon after in the district hospital due to diarrhea. Based on his interviews, Saha concludes that many thousands must have died on the wayside due to hunger and exhaustion (Page 217, (Saha, 2003)).

**3.6. Refugee mortality in Burma**

Though the majority of refugees entered India, some refugees entered Burma through its border with Bangladesh (Page 77, (Chaudhuri, 1972)). It was reported that nearly 50,000 people entered Burma in May, of which over 20% i.e. 10,000 died ‘of various diseases and starvation’, as neither the government nor the UN provided help. The Burmese government closed some of the camps, further displacing the refugees. Overall it was suggested that the condition of refugees was worse in Burma, and therefore the death rate was higher.

# References

Associated Press. (1971, July 26). Pakistan Refugee Deaths Mount From Lack of Proper Foods. *The Hartford Courant*, p. 11.

Brogan, J. E. (1971, October 5). Ever darker skies over Bengal. *The Observer*, p. 5.

Chaudhuri, K. (1972). *Genocide in Bangladesh.* Bombay: Orient Longman.

Coughlin, W. J. (1971, June 14). West Bengal Cholera Epidemic Abating: Threat to Nearby Calcutta Checked, Officials Report. *Los Angeles Times*, p. 1.

Datta, A. (2013). *Refugees and Borders in South Asia: The Great Exodus of 1971.* New York, NY, USA: Routledge.

Foster, J. (1971, June 22). 600,000 of 5 Million Refugees Have Died. *The Washington Daily News*, p. 25.

Gerlach, C. (2012). *Extremely Violent Societies: Mass Violence in the Twentieth-Century World.* Cambridge: Cambridge University Press.

Malik, A. (1972). *The Year of the Vulture.* New Delhi: Orient Longmans.

Mamoon, M., & Haq, A. M. (2007). *Media and the Liberation War of Bangladesh.* Dhaka: Ananya.

Mercer, A. (1992). Mortality and morbidity in refugee camps in eastern Sudan: 1985-90. *Disasters, 16*, 28-42.

Ministry of External Affairs, India. (1971). *Bangla Desh Documents.* New Delhi: Ministry of External Affairs.

PTI. (1971, October 1). Floods uproot DPs from new 'homes'. *The Times of India*, p. 7.

Reuters. (1971, September 14). Floods Halt Deliveries of Refugees' Supplies. *Los Angeles Times*, p. A5.

Saar, J. (1971, June 18). Pakistan Refugees Endure Chaos and Cholera: Faces Emptied of All Hope. *Life Magazine, 70*(23), pp. 22-29.

Saha, K. C. (2003). The Genocide of 1971 and the Refugee Influx in the East. In R. Samaddar, *Refugees and the State: Practices of Asylum and Care in India, 1947 - 2000* (pp. 27-28). SAGE Publications Pvt. Ltd.

Schanberg, S. (1971, October 7). How the Refugee Flood Perils India. *Chicago Tribune*, p. 22.

Schanberg, S. (1971, Sep 30). Refugee Children in India: 'Thousands' Die. *New York Times*, p. 10.

Seaman, J. A. (1972). Relief Work In a Refugee Camp for Bangladesh Refugees in India. *The Lancet, 300*(7782), 866-870.

Swaminathan, M. C., Vijayaraghavan, K., & Rao, D. H. (1983). Nutritional Status of Refugees from Bangla Desh. *Indian Journal of Medical Research, 61*, 278-284.

The Comptroller General of The United States. (1972). *United States assistance for Pakistani refugees in India.* Whasingon D.C.: Agency for International Development, Department of State, The United States.

Toole, M. J., & Waldman, R. J. (1988). An analysis of mortality trends among refugee populations in Somalia, Sudan, and Thailand. *Bulletin of the World Health Organization, 66*(2), 237-247.

United Nations High Commissioner for Refugees. (1972). *A Story of Anguish and Action: The United Nations Focal Point for Assistance to Refugees from East Bengal in India.* Geneva: Office of the United Nations High Commissioner for Refugees.

United Nations High Commissioner for Refugees. (2000). *The State of The World’s Refugees 2000: Fifty Years of Humanitarian Action.* Oxford: Oxford University Press.

United News of India. (1971, June 20). Fresh Cholera Outbreak in Bengal Camps. *The Times of India*, p. 5.
