# Supplementary material for: Death toll among the Bangladeshi refugees of the 1971 war
Source: PLoS One. 2025 Apr 4;20(4):e0320760. doi: 10.1371/journal.pone.0320760 (PMC11970699; doi:10.1371/journal.pone.0320760)
Supplement: S4 Text — (DOCX) [file pone.0320760.s004.docx]

**S4 Text: The counter-narrative by Pakistan disputing Indian refugee count**

The main body of our work rests on calculating the death rate obtained from camps and projecting it to the total death by using the total refugee counts as provided by the Govt. of India. The total refugee count, nearly 10 million, is an enormous number – it meant that nearly 15% of Bangladesh’s 70 million population had crossed over to India. Naturally, this created an unprecedented crisis in terms of food, medicine and other essential supplies, and India was spending more per month to care for the refugees than the total amount of aid UNHCR had provided (Ministry of External Affairs, India, 1971) (Volume 2, Page 88-91). However, we should note the fact that Pakistan vehemently opposed this refugee count that India had provided, claiming that India is exaggerating the number for political and financial gains. They instead officially suggested a refugee number of two million, which is much lower than the Indian estimate. Though, informally, it was reported that the Pakistani General Rao Farman Ali admitted the number of six million refugees, while President Yahya Khan admitted four million (Page 137, (Gerlach, 2012)).

(Chen & Rohde, 1973, p. 199) explained the official stance by suggesting “some have attributed this distortion to the fact that Pakistan did not recognize the Hindu refugees (who numbered about 8 million) as legitimate citizens of the Muslim state of Pakistan.” Similarly (Rummel, 1998, p. 154) suggested “These deaths resulted directly from these pitiful people, largely Hindus, fleeing for their lives before the murderous Pakistan Army.” These suggestions are likely based on the observations that Hindus constituted the majority of the refugee population, estimated to be between 70-90% (Foreign Relations of the United States, U. S. State Department, 1971) (Saha, 2003, p. 217) (Gerlach, 2012, p. 137), and it was reported that the Pakistan army indiscriminately targeted Hindus (Mascarenhas, 1971) (Schanberg, 1971), even opining that all Bengalis are Hindu at heart (Zeitlin, 1971) (Gerlach, 2012, p. 145).

Antara Datta cites a conversation between the president of Pakistan Yahya Khan and Frank L. Kellogg, the-then U. S. Secretary of State's Special Assistant for Refugee and Migration Affairs, in which Yahya declined to believe that millions of refugees were moving from East Pakistan. While the American policy was to be supportive of Pakistan, and Kellog shared the standpoint, he explained to Yahya that his personal experience too supports the Indian numbers, as he personally interviewed several refugees who seemed too ‘agriculturist’ and ‘unsophisticated’ to fabricate or parrot the same story in tandem. But Yahya dismissed this saying “all Bengalis looked the same” and the refugees are in fact destitutes brought from Calcutta (Page 22-23, (Datta, 2013)) (Central Files 1970-1973, 1971). Joseph Sisco, the U. S. Assistant Secretary of State for Near Eastern and South Asian Affairs, also privately mentioned that the Indian figures are more likely and the Pakistanis secretly admit it while denying publicly (Page 23, (Datta, 2013)) (Nixon Presidential Materials, 1971).

In face of Indian refugee registration data showing influx of millions, Yahya maintained that the Pakistani army stationed at the border have seen no refugees to leave. And the remaining refugees, that are left in India, are trying to return but being obstructed by the Indian army (Page 23, (Datta, 2013)) (Central Files 1970-73, 1971). Pakistan’s official claim was that there were only 2 million refugees in India. However, the Director General and Regional Director of UNICEF, after his visit to the refugee camps near Bongaon, described the Pakistani estimate as ‘derisory’. Based on their visiting experiences to both the border registration camps and the refugee relief camps, UNHCR as well as their High Commissioner Prince Sadruddin Aga Khan leaned towards the Indian estimates. Letters to UNHCR from the British High Commissioner in India and the UNHCR representatives in East Pakistan also corroborated this, while reporting that the Pakistanis are greatly exaggerating refugee return numbers (Page 25-26, (Datta, 2013)) (Foreign and Commonwealth Office, 1971).

While Pakistan officially played down the refugee numbers, they were claiming that a substantial number of refugees returned during the ongoing war, which too was disputed by independent foreign observers (Page 137, (Gerlach, 2012)). A foreign relief worker situated in Bangladesh at that time was quoted as saying about the Pakistani claims: “We no longer bother to visit the Government's sixty or so refugee reception centers. It’s obvious they aren’t coming back in more than a tiny trickle — none in some areas.” Commenting further that it was actually Pakistan arranging fictitious refugees, he added: “At one place we discovered the Government had a staff of professional refugees that they brought out whenever visitors came to show that something was going on.” Another foreign expert commented: “The army authorities tell you there are 2,000 refugees at some camps. You go there and find a handful of people wandering around, and under continued questioning the authorities agree maybe there are only 200. After hearing as many deceptions as we do, it quickly reaches the point at which we cannot take the Pakistan Government’s word for anything, however trivial.” (Browne, 1971).

In short, while Pakistan wanted to question both India’s political credibility and financial need of support by throwing a challenge to the refugee numbers, few believed in their claim. India maintained a relatively well-documented registration system for the refugees at the borders, and international visitors having access to the relief camps had first-hand experience of the difficult conditions. Hence all UN or other publications used the refugee count of 10 million provided by India (United Nations High Commissioner for Refugees, 2000), disregarding the Pakistani estimate entirely.

# References

Browne, M. W. (1971, October 14). Horrors of East Pakistan Turning Hope Into Despair. *New York Times*, p. 1.

Central Files 1970-1973. (1971, June 28). Telegram from the Embassy in Pakistan to the Department of State Islamabad. SOC 10 PAK, National Archives at College Park, MD.

Central Files 1970-73. (1971, August 20). Telegram No. 8534 from the Embassy in Pakistan to the US State Department. SOC 10 PAK, National Archives at College Park, MD.

Chen, L. C., & Rohde, J. E. (1973). Civil war in Bangladesh: Famine averted? In L. C. Chen, *Disaster in Bangladesh* (pp. 190-205). Oxford: Oxford University Press.

Datta, A. (2013). *Refugees and Borders in South Asia: The Great Exodus of 1971.* New York, NY, USA: Routledge.

Foreign and Commonwealth Office. (1971, October 27). Letter from P. S. Grattan, FCO to J. S. Moon, 10 Downing Street. *Relief for Pakistani refugees in India from United Nations agencies(FCO 37/965)*. The National Archives, Kew.

Foreign Relations of the United States, U. S. State Department. (1971). South Asia Crisis, 1971. *XI*, 165. Retrieved February 24, 2015, from https://history.state.gov/historicaldocuments/frus1969-76v11/pg_165

Gerlach, C. (2012). *Extremely Violent Societies: Mass Violence in the Twentieth-Century World.* Cambridge: Cambridge University Press.

Mascarenhas, A. (1971). *Genocide.* London, UK: The Sunday Times. Retrieved February 23, 2015, from http://www.cbgr1971.org/files/SundayTime/GenocideAnthonyMascarenhasDocx.pdf

Ministry of External Affairs, India. (1971). *Bangla Desh Documents.* New Delhi: Ministry of External Affairs.

Nixon Presidential Materials. (1971, July 23). Minutes of Senior Review Group Meeting. NSC Files, NSC Institutional, Files (H-Files), Box H-112, National Archives at College Park, MD.

Rummel, R. J. (1998). *Statistics of Democide: Genocide and Mass Murder Since 1900.* Münster: LIT Verlag.

Saha, K. C. (2003). The Genocide of 1971 and the Refugee Influx in the East. In R. Samaddar, *Refugees and the State: Practices of Asylum and Care in India, 1947 - 2000* (pp. 27-28). SAGE Publications Pvt. Ltd.

Schanberg, S. (1971, May 22). Bengali Refugees in Squalor in India. *New York Times*, p. 1.

United Nations High Commissioner for Refugees. (2000). *The State of The World’s Refugees 2000: Fifty Years of Humanitarian Action.* Oxford: Oxford University Press.

Zeitlin, A. (1971, July 11). Villages Wiped Out as East Pakistan Carnage Continues. *The Hartford Courant*, p. 8.
