# Supplementary material for: Death toll among the Bangladeshi refugees of the 1971 war
Source: PLoS One. 2025 Apr 4;20(4):e0320760. doi: 10.1371/journal.pone.0320760 (PMC11970699; doi:10.1371/journal.pone.0320760)
Supplement: S5 Text — (DOCX) [file pone.0320760.s005.docx]

**S5 Text:** **Refugee population composition and mortality rate by age**

The composition of the refugee population according to age and sex varied substantially between camps and over time. It was suggested that at certain times the refugee influx consisted more of vulnerable people such as children, pregnant women and mothers, and elderly people, as some adult men were staying back in Bangladesh to fight the liberation war, or were killed (Volume 18, Page 13, and Volume 11, Page 139, (Mamoon & Haq, 2007)). Also, about 70,000 adult men went back to Bangladesh from the refugee camps to participate in the liberation war (Associated Press, 1971).

There was a huge variation in mortality between the various age groups (Schanberg, 1971) (Seaman, 1972), so it is important to assess the age composition of the refugee population.

However, age-stratified death rates among the refugees are not generally available, the only source being the survey conducted by (Page 869, (Seaman, 1972)). Still, it is relevant to study the age composition of the refugee population and compare it to the age composition of the general population of Bangladesh prior to the war, to see if we can expect a difference between the death rates due to difference in composition only. A number of sources present the age decomposition of the refugee population, even though the upper limit of the age range representing ‘children’ varies from 8 to 10 years among different sources. The data sources are:

- The entire refugee population in India, and for the state of West Bengal, containing the largest refugee population, at the end of November 1971 (Page 96, (Chaudhuri, 1972)),
- A survey on the Salt Lake camp, near Calcutta, in West Bengal (Seaman, 1972),
- A survey on 3 refugee camps near Calcutta, in West Bengal (Swaminathan, Vijayaraghavan, & Rao, 1983),
- A survey on the Matlab Bazar area of Bangladesh, for 5 years prior to the war, 1966-71 (Curlin, Chen, & Hussein, 1976).

The numbers are presented in the figure below:

Interestingly, some of the records seem to suggest that the refugee population contained slightly fewer children by proportion than the general population of Bangladesh, contrary to the trend of influx seen at times. It has been attributed to the difficulty of making such long journeys in perilous conditions with young children, and the higher mortality faced by children during such journeys (see S10 Text and section 3.5 in S3 Text) (Page 137, (Gerlach, 2012)).

A more detailed decomposition by age is available for the Salt Lake camp, with strata-specific mortality rates (combining both sexes), which can be compared to equivalent numbers for the Matlab Bazar area of Bangladesh prior to the war. The table and figures below show that while the population decomposition by age is very similar, the crude death rate (CDR) was much higher among the refugees than the general population of Bangladesh.

|  | % of Population | | Crude death rate (CDR) | |
| --- | --- | --- | --- | --- |
| Age | Salt Lake camp, 1971 | Matlab Bazar, Bangladesh, 1966-71 | Salt Lake camp, 1971 | Matlab Bazar, Bangladesh, 1966-71 |
| 0-1 | 3.0% | 3.7% | 553.8 | 127.6 |
| 1-4 | 9.0% | 12.7% | 212.1 | 25.7 |
| 4-8 | 10.1% | 15.7% | 49.9 | 3.7 |
| Adult | 74.8% | 64.9% | 3.4 | 5.3 |
| Very old (65+) | 3.2% | 3.0% | 238.4 | 73.2 |

The aggregate CDR in Matlab Bazar prior to the war was 15, less than the national average of 17. An age-adjusted CDR for the population of Bangladesh, using either the all-India refugee age distribution, or the Salt Lake age distribution, would give a slightly lower value of overall CDR (approximately 13). Therefore, by using the CDR of 17 as our baseline to calculate expected mortality among the refugees, which is then subtracted from the observed mortality, the excess death count is underestimated.

# References

Associated Press. (1971, December 1). Mrs. Gandhi Urges Free East Pakistan. *Los Angeles Times*, p. 6.

Chaudhuri, K. (1972). *Genocide in Bangladesh.* Bombay: Orient Longman.

Curlin, G. T., Chen, L. C., & Hussein, S. B. (1976). Demographic Crisis: the impact of the Bangladesh civil war (1971) on births and deaths in rural area of Bangladesh. *Population Studies: A Journal of Demography, 30*(1), 87-105.

Gerlach, C. (2012). *Extremely Violent Societies: Mass Violence in the Twentieth-Century World.* Cambridge: Cambridge University Press.

Mamoon, M., & Haq, A. M. (2007). *Media and the Liberation War of Bangladesh.* Dhaka: Ananya.

Schanberg, S. (1971, Sep 30). Refugee Children in India: 'Thousands' Die. *New York Times*, p. 10.

Seaman, J. A. (1972). Relief Work In a Refugee Camp for Bangladesh Refugees in India. *The Lancet, 300*(7782), 866-870.

Swaminathan, M. C., Vijayaraghavan, K., & Rao, D. H. (1983). Nutritional Status of Refugees from Bangla Desh. *Indian Journal of Medical Research, 61*, 278-284.
