# Supplementary material for: Death toll among the Bangladeshi refugees of the 1971 war
Source: PLoS One. 2025 Apr 4;20(4):e0320760. doi: 10.1371/journal.pone.0320760 (PMC11970699; doi:10.1371/journal.pone.0320760)
Supplement: S6 Text — (DOCX) [file pone.0320760.s006.docx]

**S6 Text:** **Adjustment for variance inflation due to stratification on age**

As seen in S5 Text, mortality rates are not same for all refugees – children bear the brunt of it mostly (Toole & Waldman, 1988). Seaman (Seaman, 1972) showed that for the Salt Lake camp 76% of the total mortality occurred in children less than 8 years of age. He also observed that malnutrition is 4 times higher in children (age < 5 years) compared to the adults, which killed hundreds of children each day around camps in West Bengal during September when the problem was particularly severe (Schanberg, 1971). In Banjetia and Lalbagh camp too majority of the deceased were children (Page 184, (Osmani, 2012)). The deputy commissioner of the Goalpara district in Assam mentioned the same point in an interview to K. C. Saha about Chapor camp (Page 221-222, (Saha, 2003))**.** Seaman from his November survey in Salt Lake camp on 4770 people presented age-stratified mortality rates (Seaman, 1972), the only such available data for this refugee population:

| **Age band** | **Population** | **% of population** | **# Deaths** | **Death rate (%)** | **Variance** |
| --- | --- | --- | --- | --- | --- |
| 0 to 1 | 138 | 2.8 | 33 | 23.9 | 25.1 |
| 1 to 4 | 430 | 9 | 38 | 8.8 | 34.6 |
| 4 to 8 | 481 | 10 | 10 | 2.1 | 9.8 |
| ‘Adult’ | 3570 | 75 | 8 | 0.2 | 7.9 |
| ‘Very old’ | 151 | 3.2 | 15 | 9.9 | 13.5 |
| **All (ignoring strata)** | **4770** | **–** | **104** | **2.2** | **101.7** |

**6.1. Stratified estimation formulae**

Suppose strata *S_j_* in a particular refugee camp has *n_j_* individuals with a mortality rate of *p_j_* and number of deaths within that strata is estimated to be *r_j_*. Using expressions from the binomial model, we can state that *E*(*r_j_*|*S_j_*) = *n_j_p_j_*, and *V*(*r_j_*|*S_j_*) = *n_j_p_j_q_j_*, which is approximately equal to *n_j_p_j_* as *p* is very small.

We assume that people of all strata have spent the same time in the camp. This allows us to find the mean and variance of the death toll just by focusing on the mortality rate per person in the camps.

Total death toll in the camp is, *E*(*r*) = Σ*E*(*r_j_*|*S_j_*) = Σ *n_j_p_j_*. This is the aggregated average across all strata.

Variance of this total death toll is, *V*(*r*) = Σ*V*(*r_j_*|*S_j_*) + *V*(*E*(*r_j_*|*S_j_*)) = Σ(*n_j_p_j_*) + *V*(*n_j_p_j_*).

The first term is sum of strata-specific variance, also called the ‘within’ variance, and the second term is variance of strata-specific expected mean values, called the ‘across’ or ‘between’ variance (Rao, 2008).

**6.2. Calculating variance of stratified data**

The variance calculated in the last row of the table above is the variance of the total mortality following the binomial model assuming no effect of stratification. But as we see a huge variation in age-specific death rates across strata, we need to apply a more detailed statistical estimation approach to the overall variance. Note that the estimated total mortality is unaffected by stratification, so adjusting it for strata-specific rates is not necessary.

We follow the steps described above for calculating the total variance including variation within and across strata. The total variance is comprised of two terms. The first term is sum of strata-specific variance (rows 1-5, column 6), 91.0 in this case. The second term is variance of strata-specific death counts (rows 1-5, column 4), 151.7 in this case. Together, the total is 242.8, which is much higher than the 101.7 obtained under a binomial model with no stratification.

**6.3. Calculating an inflation factor for stratification**

We can calculate an inflation factor (a multiplying factor) as the ratio of the stratified variance to the binomial model variance.

Using the numbers above, we get an inflation factor of 242.8/101.7 = 2.387 for the variance of Salt Lake camp. As age stratified data for other camps are not available, we assume that the same inflation factor is useable for the other camps as well.

Taking its square root, the inflation factor for any estimate of standard deviation is 1.545.

# References

Osmani, W. R. (2012). *The historical background of the immigration problems in Assam 1946 to 1983.* Aligarh: Aligarh Muslim University.

Rao, R. (2008). *Linear Statistical Inference and its Applications* (2nd ed.). New York: Wiley-Interscience.

Saha, K. C. (2003). The Genocide of 1971 and the Refugee Influx in the East. In R. Samaddar, *Refugees and the State: Practices of Asylum and Care in India, 1947 - 2000* (pp. 27-28). SAGE Publications Pvt. Ltd.

Schanberg, S. (1971, Sep 30). Refugee Children in India: 'Thousands' Die. *New York Times*, p. 10.

Seaman, J. A. (1972). Relief Work In a Refugee Camp for Bangladesh Refugees in India. *The Lancet, 300*(7782), 866-870.

Toole, M. J., & Waldman, R. J. (1988). An analysis of mortality trends among refugee populations in Somalia, Sudan, and Thailand. *Bulletin of the World Health Organization, 66*(2), 237-247.
