# Supplementary material for: Death toll among the Bangladeshi refugees of the 1971 war
Source: PLoS One. 2025 Apr 4;20(4):e0320760. doi: 10.1371/journal.pone.0320760 (PMC11970699; doi:10.1371/journal.pone.0320760)
Supplement: S7 Text — (DOCX) [file pone.0320760.s007.docx]

**S7 Text:** **Descriptions** **of refugee camps’ death tolls**

Below we discuss the various sources which constitute records of mortality among the various camps and were used to construct the aggregated estimate of total death toll. These data are summarized in Table 2, and the camp locations are shown in Figure 2.

**7.1. Salt Lake Refugee Camp, West Bengal**

Salt Lake refugee camp had a total population of 170,000 (Seaman, 1972). Seaman conducted a survey on a sample population of 4,770 during October-November 1971, drawing from the list of people admitted to the camp hospital during July-November. The survey reports a death toll of 103 among participants. This death toll, extrapolated to total camp population, is 3671 according to the author.

This excludes, as the author notes, deaths due to the cholera epidemic in June, after which the camp was largely inoculated and therefore cholera incidence was low during the survey period (Seaman, 1972).

However, we have a report for Salt Lake camp during the early days of the June cholera epidemic: 1250 deaths by June 9 according to Govt. of India (Datta, 2013, p. 148).

**7.2. Itkhola Refugee Camp, West Bengal**

A political party report on 13^th^ June 1971 described the Itkhola refugee camp in Bangaon district, near Calcutta, to be a small ‘model’ camp, as run by the party (Mamoon & Haq, 2007, pp. 58, Volume 18), which was notably successful in keeping the epidemics in check till then due to inoculations and improved sanitary practices. Among the 18,000 refugees, only 17 had died till then, primarily from diarrhea. Mortality estimates from other factors were not available.

**7.3. Karimpur Refugee Camp, West Bengal**

Karimpur camp’s information was provided in a news article (Saar, 1971). The article mentions 700 death toll among 15,000 camp population, due to a severe epidemic of cholera. It was published on June 18, 1971. Hence, we use June 17, 1971 to be the report date of this death toll. However, Saar reports that the camp was already abandoned by then, due to high incidence of cholera. So, our timespan for the camp is an over-estimate, and therefore its death rate is an under-estimate. The start date of the camp was not reported. However, as for the second set of data for the Salt Lake camp, these death figures are from a monsoon-specific cause, cholera. So for this and several other camps, the start date of the period of consideration towards the analysis was taken to be 1^st^ June (see S2 Text for further explanations).

**7.4. Boyra Refugee Camp, West Bengal**

(Coggin, 1971) cite a German physician, Dr. Mathis Bromberger, working at the camp, to report that about 5000 people died in the camp due to cholera. The report was published on 2^nd^ August 1971. The total camp population at that time, or the number of deaths due to other causes were not available. However, (Lescaze, 1971) reports on 26^th^ April that the total refugee population of camps around Boyra was 40,000.

**7.5. Kalyani Refugee Camp, West Bengal**

500 people died from cholera according to official reports during the month of June in a large refugee camp near Kalyani (Chaudhuri, 1972, p. 92). While refugee numbers in that specific camp is not available, it was reported that several camps near Kalyani had a total refugee population of about 100,000, while the largest camp had about 50,000 refugees (Saha, 2003, p. 217). We use the largest camp’s population count here, to ensure a conservative estimate.

**7.6. Refugee Camps in Nadia District, West Bengal**

1,000 refugees were reported to have died from cholera in the Nadia district of West Bengal, from a news report on 5^th^ June 1971 quoting the state health minister. The report mentioned 250,000 refugees in total crossing into the Nadia district, though they may have been dispersed to other nearby districts too. Further specific details, or information on mortality from other factors were not available (Mamoon & Haq, 2007, pp. 119-121, Volume 11). We use the entire 250,000 number to ensure a conservative estimate.

**7.7. Barasat Jail Compound Refugee Camp, West Bengal**

The information of Barasat jail compound refugee camp comes from a survey (Gardner, Rohde, & Majumdar, 1972, p. 834). This survey mentions a death toll of 25 among 453 survey participants from March 25, 1971, to October 27, 1971. Barasat camp had a population of 3,000. We extrapolated the death toll among survey participants to total refugee population.

**7.8. Barasat Hospital and nearby Refugee Camp, West Bengal**

The Barasat hospital serving a nearby refugee camp of 7,000 refugees reported on 26^th^ July that about 10 refugees die every day in the hospital, mostly children from starvation (Associated Press, 1971). Seaman reported upon surveying the Salt Lake camp that for each death in the associated hospital, 4 more deaths occurred in the camp (Seaman, 1972). Therefore, the conversion factor between a camp and its associated hospital is 4+1=5, and by extrapolation, the total death rate is considered to be 10×5 = 50 per day. As the report was published on 26^th^ July, the period of this death rate is taken to be 1-25 July. When death rates are provided but a specific duration is not provided, we take the month (till the reporting date when available) to be the period for which the death rate was applicable.

**7.9. Banjetia and Lalbagh Refugee Camp, West Bengal**

K. C. Saha mentions “about a thousand” death toll out of 20,000 camp population in Banjetia and Lalbagh refugee camp (Saha, 2003, p. 218). He gets this information from S. S. Chattopadhyay, the additional district magistrate of Murshidabad, who was quoted saying, "Immediately after the independence of Bangladesh the refugees just melted away and repatriated of their own will.” The independence war of Bangladesh ended on December 16, 1971. Hence, we consider December 16, 1971, to be the report date of this camp’s death toll, and in other cases where an ending date is not available.

The camp was established “in the summer months” due to a heavy influx of refugees, when the monsoon rains had started (Saha, 2003, p. 214). So, June 1 was taken to be the camp starting date. The period is kept as wide as possible to ensure a conservative estimate.

**7.10. Balat & Mailam Refugee Camp, Meghalaya**

According to A. Dutta, the Balat and Mailam camps had 21,000 refugees till Mid-September. After Mid-September 1971, a new lot of 150,000 refugees arrived. Due to the sudden arrival of so many refugees, cholera spread rapidly. The author mentions 100-150 refugee dying from cholera during that time (Datta, 2013, p. 108). Therefore, the average rate of 125 deaths per day is used, and the period is taken to be 15-30 September.

**7.11. My Long Refugee Camp, Meghalaya**

The My Long camp, situated in the Khasi-Jaintia hills of Meghalaya, was reported to have a total population of 80,000 (Chaudhuri, 1972, p. 77). About 2500 are said to have died from cholera in May-June, while 1000 people died of pneumonia. Deaths due to other causes were not available.

The camp name of My Long is not seen with that spelling in other records; however, the Indian Government’s list of refugee camps contains a similar name in the same area, Myilliem (Ministry of External Affairs, India, 1971). We therefore use this location while preparing the map.

**7.12. Ampati Refugee Camp, Meghalaya**

Colonel Zahir, a Bangladeshi freedom fighter, wrote from his personal experience about Ampati and the surrounding camps (Zahir, 2016). These in total had a refugee population of 45,000, of which 3,500 were said to have died, to a large extent due to monsoon and epidemics. Based on his conversations with the people, trying to make a list of the people who died, “the number of 3,500 dead was obtained from the refugees, families of the dead and the local people of Ampati and surrounding areas.” The refugees started to arrive towards the end of April, and the camp continued till the end of the war. We therefore take 1^st^ May to be the beginning of the period.

**7.13. Chapor Refugee Camp, Assam**

M.P. Bezbaruah, then Deputy Commissioner of Goalpara district of Assam, mentioned in an interview that a refugee camp at Chapor near the town of Dhubri was set up with 50,000 refuges (Saha, 2003, p. 221). 500 refugees, mostly children, had died in the camp according to official records. But no dates were available for either of the numbers. Therefore, the possible broadest duration, 1^st^ May to 16^th^ December, was taken to be the reporting period to ensure a conservative estimate.

**7.14. Jambu Island, Orissa**

A severe cyclone hit the coastal areas of Eastern India on 29^th^ October. The destruction was described as ‘catastrophic’ and ‘complete’, with entire villages washed away (November 2) (Associated Press, 1971). Immediately after the cyclone, 15-20,000 people were estimated to have died, while the coastal island town of Jambu was reported to have suffered 2,500 fatalities according to official reports, though unofficial reports suspected higher deaths. Further deaths were expected from subsequent flooding, starvation, and cholera, as the possibility of providing aid was very slim. Orissa was said to have been particularly lacking in infrastructure.

A case report on the cyclone by an US Government Agency reported that the entire Jambu island had a total population of 33,000, consisting primarily of refugees (Agency for International Development, 1972, p. 1). In particular, the town of Jambu on the island had a total population of 4,500, of which one-third i.e. 1,500 were reported dead from the cyclone. A later assessment on November 7 (UPI, 1971) updated the death estimate in Jambu island to 5,000. It also reported on another village, Suniti, with a population of 2,000, of which 3/4^th^ i.e. 1,500 people reportedly died.

While the exact number of refugees on the island or specifically the number of dead refugees among the dead people were not known, following the US Government case report we are using the entire data of the Jambu island to estimate the death rate, as it was suggested that the island consisted primarily of refugees. In any case, it is a reasonable assumption that the death rate would have been similar among the refugees and the residents, if not higher. It was not reported when the refugees started to move to Jambu, or the mortality rates for causes other than the cyclone. The only information available in this regard was that the Chief Minister of Orissa decided to accept refugees to be placed in the coastal areas on June 19^th^ (Datta, 2013, p. 141).

**7.15. Mana Refugee Camp, Madhya Pradesh (presently Chhattisgarh)**

The Mana camp was reported to be the biggest refugee camp outside the border states. The process of moving refugees there to ease the pressure on refugee camps in the border states started on 3^rd^ June (Datta, 2013, pp. 141-143). A report on July 4^th^ mentioned that by June 21, 52,000 refugees arrived there, in addition to 85,000 people already living there by then (Joshi, 1971). It was also reported that 80 refugees have died since the new arrivals. The causes of death, according to the report, was “mostly because of general weakness, and not cholera, according to the authorities.” The period of consideration was therefore taken to be 21^st^ June to 3^rd^ July.

A subsequent report on 21^st^ September 1971 mentioned that daily 29 to 49 people were dying due to cholera and gastroenteritis, during a severe bout of the epidemic. The camp population was 60,000 then (Mamoon & Haq, 2007, pp. 109, Volume 18). 1-21 September was therefore taken to be the period of consideration, and the average rate of 39 deaths per day was therefore extrapolated to this period to obtain the total number of deaths.

# References

Agency for International Development. (1972). *Case Report: India - Cyclone.* Washington, DC.

Associated Press. (1971, November 2). Cyclone, Wave Kill Thousands in India. *Los Angeles Times*, p. A1.

Associated Press. (1971, July 26). Pakistan Refugee Deaths Mount From Lack of Proper Foods. *The Hartford Courant*, p. 11.

Chaudhuri, K. (1972). *Genocide in Bangladesh.* Bombay: Orient Longman.

Coggin, D. (1971, August 2). Pakistan: The Ravaging of Golden Bengal. *Time*.

Datta, A. (2013). *Refugees and Borders in South Asia: The Great Exodus of 1971.* New York, NY, USA: Routledge.

Gardner, P., Rohde, J. E., & Majumdar, M. B. (1972). Health Priorities Among Bangladesh Refugees. *The Lancet, 299*(7755), 834-836.

Joshi, V. T. (1971, July 4). The Times Weekly Special Issue. *The Times of India*, p. A2.

Lescaze, L. (1971, April 26). Bengali Refugees Fill Indian Camps. *The Washington Post*, p. A10.

Mamoon, M., & Haq, A. M. (2007). *Media and the Liberation War of Bangladesh.* Dhaka: Ananya.

Ministry of External Affairs, India. (1971). *Bangla Desh Documents.* New Delhi: Ministry of External Affairs.

Saar, J. (1971, June 18). Pakistan Refugees Endure Chaos and Cholera: Faces Emptied of All Hope. *Life Magazine, 70*(23), pp. 22-29.

Saha, K. C. (2003). The Genocide of 1971 and the Refugee Influx in the East. In R. Samaddar, *Refugees and the State: Practices of Asylum and Care in India, 1947 - 2000* (pp. 27-28). SAGE Publications Pvt. Ltd.

Seaman, J. A. (1972). Relief Work In a Refugee Camp for Bangladesh Refugees in India. *The Lancet, 300*(7782), 866-870.

UPI. (1971, November 7). Cyclone wrecks islands. *Times News*, p. 32.

Zahir, L. C. (2016). Ampati, a graveyard and the benevolent woman. In M. G. Cardozo, *In Quest of Freedom: The War of 1971 - Personal Accounts by Soldiers from India and Bangladesh.* New Delhi: Bloomsbury.
