# Supplementary material for: Death toll among the Bangladeshi refugees of the 1971 war
Source: PLoS One. 2025 Apr 4;20(4):e0320760. doi: 10.1371/journal.pone.0320760 (PMC11970699; doi:10.1371/journal.pone.0320760)
Supplement: S8 Text — (DOCX) [file pone.0320760.s008.docx]

**S8 Text: Potential adjustment of official death counts**

**8.1. Official under-reporting of deaths in refugee camps**

Often deaths were under-reported in refugee camps due to various reasons, as is common in such crisis situations (Dick & Simmonds, 1983). Mortality during peak epidemic periods was extremely high, and government workers could not cope with the volume of the sick and dead people. Most of the hospitals were full to the brim, and there were many deaths outside of the hospitals. It was said the recorded death rates would’ve been higher if there was more space to treat patients: “As soon as their bodies are removed, more refugees are brought to the hospital to be treated—and, in many cases, to die.” (Associated Press, 1971). (Seaman, 1972) also reported upon surveying the Salt Lake camp that for each death in the associated hospital, 4 more deaths occurred in the camp, which would usually go unrecorded. “They are dying in such numbers we can’t even keep count”, said a relief worker (Saar, 1971).

Official estimates usually only count bodies seen by an official, either in the hospital or during visits. But often dead bodies were missed from counting, intentionally or unintentionally. (Seaman, 1972) reported upon surveying the Salt Lake camp that for each death in the associated hospital, 4 more deaths occurred in the camp, which would usually go unrecorded. But officials and doctors also intentionally under-reported the official mortality figures at times, as they felt the deaths might indicate they are doing a poor job. A spokesman of the West Bengal Health Services suggested that the reported numbers are probably deflated, because the doctors and officials think high death tolls might paint them as incompetent or hold them responsible for the deaths (Page 92, (Chaudhuri, 1972)). In other situations, they underreported to not scare the refugees (Page 106, (Datta, 2013)). In an interview, a former govt. employee in charge of refugee records for West Bengal suggested that there was govt. pressure for keeping the death numbers low (Page 20 (Chowdhury, 2019)).

It was also alleged by Indian officials that refugees would often hide the dead bodies without reporting, in order to continue receive the supplies allocated to that person (Sabharwal, 1971) (Brogan, 1971) (Schanberg, 1971). At times, dogs or vultures would eat away the dead bodies without them being counted (Page 92, (Chaudhuri, 1972)).

This informs us that official figures for deaths during peak epidemic periods are underestimates which we might want to adjust for.

**8.2. Calculation of a conversion factor using cholera mortality records**

Among the epidemics, cholera was one of the most severe and widely reported, and therefore we have several news reports presenting both official and unofficial counts over a span of time, which allow us to make a comparison of the two. The incidence of cholera began in early May, but became really severe at the beginning of June when monsoon arrived in full form (Page 146, (Datta, 2013)) (Page 72, (Chaudhuri, 1972)) (Zahir, 2016). Multiple reports enable us to track the progress of the cholera death toll through the early part of its peak period, June, in West Bengal.

We track the discrepancy between official (recorded) and unofficial death estimates mentioned at the same time in published reports. As West Bengal was the focal point of the refugee crisis, sometimes it is unclear from the records whether the report is referring to all camps or only WB camps, or a specific camp. The official numbers also vary to some extent, as different state or central officials are quoted at different times. In each case, the unofficial figures were either provided by WHO, or a govt. official off the record, or by ‘sources working closely in touch with the refugee relief work’.

| **Date** | **Official Estimate** | **Unofficial Estimate** | **Inflation factor** | **Source** |
| --- | --- | --- | --- | --- |
| 5 June 1971 | 1,500 (Nadia only) | 2,500 | 1.67 | (Associated Press, 1971) |
| 6 June | 1,250 | 3000 (WHO) | 2.4 | (Reuters, 1971) |
| 6 June | 1,250 | 8000 (govt. official) | 6.4 | (Reuters, 1971) |
| 7 June | 4,000 | 10,000 | 2.5 | (Associated Press, 1971) |
| 9 June | 3600 | 5000 | 1.39 | (Schanberg, 1971) |
| 9 June | 1500 | 5000 | 3.33 | (The Times of India News Service, 1971) |
| 10 June | 2405 | 2405+2400 (govt. official) | 2.00 | (United News of India, 1971) |

Comparing the official and unofficial numbers in the same report we can calculate the inflation factor for that report, which is the factor by which the official figure has to be multiplied to obtain the unofficial death count. Information for all the reports collected in the table can be aggregated to estimate an overall inflation factor.

Since the inflation factors are multipliers, and the set contains one relatively higher value which requires the use of a more robust aggregator, we use the geometric mean instead of the simple average (arithmetic mean). The geometric mean of these inflation factors is 2.489. Using this multiplier, the 9^th^ June official estimate of 1250 deaths for the Salt Lake camp in Calcutta metropolitan area (Page 148, (Datta, 2013)) becomes 1250 × 2.489 = 3111 (rounded) unofficial deaths in total for that camp. This figure is used in Table 2 for the Salt Lake camp.

Note that, a few thousand deaths due to the Cholera epidemic among 170,000 residents was not unusual during that time. For example, On June 2, during the early days of cholera, we have Mr. A. K. Gupta, the additional district magistrate of Nadia, reporting that 600 refugees have died within his area in just the past 72 hours (Bhaumik, 1971).

# References

Associated Press. (1971, June 5). E. Pakistani Cholera Deaths Put at 5,000. *Los Angeles Times*, p. 14.

Associated Press. (1971, July 26). Pakistan Refugee Deaths Mount From Lack of Proper Foods. *The Hartford Courant*, p. 11.

Associated Press. (1971, June 7). U.S., Britain Withdrawing Diplomats From E. Pakistan: Urges Evacuation. *The Washington Post, Times Herald*, p. A8.

Bhaumik, K. (1971, Jun 3). 600 Refugees Die of Cholera. *The Times of India*, p. 1.

Brogan, J. E. (1971, October 5). Ever darker skies over Bengal. *The Observer*, p. 5.

Chaudhuri, K. (1972). *Genocide in Bangladesh.* Bombay: Orient Longman.

Chowdhury, S. Q. (2019). *Muktijuddhe bharoter chikitsha sohayta (Medical help of India in Liberation War).* Dhaka: Somoy Prakashan.

Datta, A. (2013). *Refugees and Borders in South Asia: The Great Exodus of 1971.* New York, NY, USA: Routledge.

Dick, B., & Simmonds, S. (1983). Refugee health care: Similar but different? *Disasters, 7*(4), 291-303.

Reuters. (1971, June 8). Pakistanis in India Swell to 5 Million: Refugee Deaths in Cholera Epidemic Reported at 8000. *Los Angeles Times*, p. 1.

Reuters. (1971, June 8). Refugees and Cholera Increase in India. *New York Times*, p. 3.

Saar, J. (1971, June 18). Pakistan Refugees Endure Chaos and Cholera: Faces Emptied of All Hope. *Life Magazine, 70*(23), pp. 22-29.

Sabharwal, P. (1971, October 14). India expects 12 million refugees by '72. *The Sun*, p. A8.

Schanberg, S. (1971, June 9). Disease, Hunger and Death Stalk Refugees Along India's Border. *New York Times*, p. 3.

Schanberg, S. (1971, October 7). How the Refugee Flood Perils India. *Chicago Tribune*, p. 22.

Seaman, J. A. (1972). Relief Work In a Refugee Camp for Bangladesh Refugees in India. *The Lancet, 300*(7782), 866-870.

The Times of India News Service. (1971, June 10). Killer cholera outbreak feared. *The Times of India*, p. 1.

United News of India. (1971, June 20). Fresh Cholera Outbreak in Bengal Camps. *The Times of India*, p. 5.

Zahir, L. C. (2016). Ampati, a graveyard and the benevolent woman. In M. G. Cardozo, *In Quest of Freedom: The War of 1971 - Personal Accounts by Soldiers from India and Bangladesh.* New Delhi: Bloomsbury.
