# Supplementary material for: Death toll among the Bangladeshi refugees of the 1971 war
Source: PLoS One. 2025 Apr 4;20(4):e0320760. doi: 10.1371/journal.pone.0320760 (PMC11970699; doi:10.1371/journal.pone.0320760)
Supplement: S9 Text — (DOCX) [file pone.0320760.s009.docx]

**S9 Text: Other incomplete information about refugee deaths that could not be included in the analysis**

In the refugee camps in the Khasi-Jaintia hills of Meghalaya, nearly 10,000 refugees were reported to have died from cholera, malnutrition, gastroenteritis, and exposure to rains between May and July (Pages 72-73, (Chaudhuri, 1972)). But the total population in these camps were not available.

In the Bakchara camp of West Bengal, a journalist reported on 24^th^ July 1971 that the camp suffered a severe bout of gastrocolitis for the last five days. While no records were being kept of the dead, he spoke with some of the refugee families to report that he heard 8 children had died in the camp among these families. Another person reported that 5 of his adult family members were admitted to a nearby hospital outside of the camp, where 3 of them died. But a total count of adult refugee deaths in the camp for that period was not available. The camp began on 11^th^ May, and the population then was 5,558. (Volume 6, Page 102, (Mamoon & Haq, 2007)). But camp population during the period of deaths was not available either.

It was estimated in November that 4,300 refugee children were dying per day (UPI, 1971).

A report from the Reuters in mid-September mentioned the devastation brought about by the monsoon floods. It reported that 350,000 refugees were ‘marooned’ by the floods. PTI reported that 300 refugees had died of cholera in Assam, but unofficial figure was more than 500. In North Bengal 1,300 refugees were estimated to have died from the flood (Reuters, 1971).

Often the firing or assault by Pakistani army on the refugees during their migration did not cause immediate death, but the refugees subsequently died on the roadside or soon upon arrival at the camp (Schanberg, 1971). A news report on 16^th^ September mentioned 504 refugees being admitted in three hospitals near Calcutta with severe injuries due to Pakistan army assaults, and despite their best medical efforts 10 people died (Volume 18, Page 106, (Mamoon & Haq, 2007)). Another report on 17^th^ September of 27 such wounded refugees admitted to a hospital mentioned 1 dead (Volume 18, Page 107, (Mamoon & Haq, 2007)).

The effects of war sometime spilled into the refugee camps. On some occasions, the Pakistani army even fired at refugee camps inside the Indian border, causing deaths and injuries (Schanberg, 1971) (Volume 6, Page 118; and Volume 18, Page 108, (Mamoon & Haq, 2007)). It also included the acts of Pakistani ‘saboteurs’ – in one instance, 4 refugees in the Balat camp died when the camp was set on fire by Pakistani agents, according to the official spokesperson (The Times of India News Service, 1971).

Antara Datta cites social worker Anjali Lahiri who worked in refugee camps in the state of Meghalaya. Reporting from Sela camp, she notes that when faced with a lack of cooking oil the refugees collected and used car engine oil for cooking. She further comments that this was a widespread practice due to food shortages, and 80 refugees died from eating such adulterated oil in Amlareng camp (Page 109, (Datta, 2013)).

Clashes between refugees, who were protesting about lack of food supplies, and police in several places left 6 dead (Sabharwal, 1971). A similar protest in the Mana camp led to police firing upon the protesting refugees due to violent clashes, which led to 2 deaths (Volume 6, Page 102, (Mamoon & Haq, 2007)). In another incident, 3 refugees died from police fire in the Manugunj camp of Jalpaiguri, West Bengal (Volume 18, Page 100, (Mamoon & Haq, 2007)).

While most reports of refugee deaths focused on epidemics such as cholera, it was also reported that some refugees die from other causes such as snake bites. A report in June estimated that of the 5.2 million refugees at that point in West Bengal, about 40-50 people die from such causes every week (The Times of India News Service, 1971).

# References

Chaudhuri, K. (1972). *Genocide in Bangladesh.* Bombay: Orient Longman.

Datta, A. (2013). *Refugees and Borders in South Asia: The Great Exodus of 1971.* New York, NY, USA: Routledge.

Mamoon, M., & Haq, A. M. (2007). *Media and the Liberation War of Bangladesh.* Dhaka: Ananya.

Reuters. (1971, September 14). Floods Halt Deliveries of Refugees' Supplies. *Los Angeles Times*, p. A5.

Sabharwal, P. (1971, October 14). India expects 12 million refugees by '72. *The Sun*, p. A8.

Schanberg, S. (1971, May 22). Bengali Refugees in Squalor in India. *New York Times*, p. 1.

The Times of India News Service. (1971, November 2). 'Counter-action' ends shelling of Kamalpur. *The Times of India*, p. 1.

The Times of India News Service. (1971, June 27). DP influx still continues. *The Times of India*, p. 9.

UPI. (1971, November 2). 200,000 Children Face Starvation. *The Hartford Courant*, p. 27.
