# Supplementary material for: Death toll among the Bangladeshi refugees of the 1971 war
Source: PLoS One. 2025 Apr 4;20(4):e0320760. doi: 10.1371/journal.pone.0320760 (PMC11970699; doi:10.1371/journal.pone.0320760)
Supplement: S10 Text — (DOCX) [file pone.0320760.s010.docx]

**S10 Text: Mortality among refugees within Bangladesh while journeying to and from India**

Wayside deaths also include refugees dying within Bangladesh on their way to India. These are substantial to consider but we did not use these in our estimation as these deaths did not occur within India. Mortality rates among internally displaced people are also usually several times higher than peacetime rates (Heudtlass, Speybroeck, & Guha-Sapir, 2016). There are numerous reports of accidental deaths and killings by the Pakistani army. (Lescaze, 1971) interviewed a refugee on army brutalities who said “he saw 500 dead bodies as he fled into the countryside”. In one instance, 200 refugees were killed inside Bangladesh on their way to India due to shelling by Pakistani army (Dutta, 1971). (Page 87, (Chaudhuri, 1972)) quotes a harrowing account from a PTI (Press Trust of India) report of 28^th^ July: “50 refugees fled into a jute field near the Indian border when they heard a Pakistani army patrol approaching. Suddenly a six-month old child in its mother's lap started crying. Failing to silence the child and apprehending that the refugees might be attacked, the woman throttled the infant to death.”

After the war, the Bangladesh government estimated the total number of displaced people to be 20 to 30 million (Page 139, (Gerlach, 2012)) (Page 147, (Chaudhuri, 1972)). The death rates would’ve been higher in these people due to difficult conditions including various epidemics. Epidemics were rife in refugee transit camps within Bangladesh. This also includes transit camps when refugees were returning home after the war. Over 2,500 people were reported to have died in some such transit camps due to small-pox and chicken-pox during January-February 1972 (Page 138, (Chaudhuri, 1972)). The returning refugees were carriers of epidemics, which led to a substantial increase in the incidence and death rates of several epidemics in post-war Bangladesh, with children having the highest rates (Curlin, Chen, & Hussein, 1976). “By the onset of the war, Bangladesh had been declared essentially smallpox-free with no reported cases in late 1970. But by early 1972, smallpox was again prevalent in epidemic proportions as a result of importation of the disease by the returning refugees from India.”

# References

Chaudhuri, K. (1972). *Genocide in Bangladesh.* Bombay: Orient Longman.

Curlin, G. T., Chen, L. C., & Hussein, S. B. (1976). Demographic Crisis: the impact of the Bangladesh civil war (1971) on births and deaths in rural area of Bangladesh. *Population Studies: A Journal of Demography, 30*(1), 87-105.

Dutta, S. K. (1971, April 17). 200 die in Sylhet area. *The Times of India*, p. 1.

Gerlach, C. (2012). *Extremely Violent Societies: Mass Violence in the Twentieth-Century World.* Cambridge: Cambridge University Press.

Heudtlass, P., Speybroeck, N., & Guha-Sapir, D. (2016). Excess mortality in refugees, internally displaced persons and resident populations in complex humanitarian emergencies (1998–2012) – insights from operational data. *Conflict and Health, 10*.

Lescaze, L. (1971, April 26). Bengali Refugees Fill Indian Camps. *The Washington Post*, p. A10.
